# Hospital Furniture and Furnishing

**Published:** 1907-06-29

**Authors:** 


### HOSPITAL FURNITURE AND FURNISHING.

### AN OPPORTUNITY FOR PRACTICAL OFFICIALS.

Messrs. James Shoolbred and Co., Tottenham Court Road, have furnished a few rooms in one portion of their extensive premises where examples of hospital furniture manufactured by them may be seen. Here anyone who requires articles of this description will find many patterns and obtain some useful information. Messrs. Shoolbred and Co. have supplied several Hospitals and Institutions with furniture of every description. When we visited their emporium recently, we were struck with the relative cheapness of the furniture, having regard to the quality of the goods which they manufacture. It ought to be widely known, in the interests of hospitals, that Messrs. Shoolbred are prepared to manufacture furniture from special patterns and to make up new and original hospital units, provided anyone engaged in hospital work, who has ideas, will call in Tottenham Court Road and see their representative, Mr. Frank. The opportunity here afforded to experience of joining hands with the manufacturer will have this result, that more up-to-date articles of the most suitable character may be made available for hospital purposes at the lowest possible cost. We have had dealings with Messrs. Shoolbred and Co. for more years than we care to remember, and we can testify, from experience, that anyone dealing with this firm is sure to get good value for the money expended.

